# *In silico* Prediction, *in vitro* Antibacterial Spectrum, and Physicochemical Properties of a Putative Bacteriocin Produced by *Lactobacillus rhamnosus* Strain L156.4

**DOI:** 10.3389/fmicb.2017.00876

**Published:** 2017-05-19

**Authors:** Letícia de C. Oliveira, Aline M. M. Silveira, Andréa de S. Monteiro, Vera L. dos Santos, Jacques R. Nicoli, Vasco A. de C. Azevedo, Siomar de C. Soares, Marcus V. Dias-Souza, Regina M. D. Nardi

**Affiliations:** ^1^Departamento de Biologia Geral, Instituto de Ciências Biológicas, Universidade Federal de Minas GeraisBelo Horizonte, Brazil; ^2^Departamento de Microbiologia, Instituto de Ciências Biológicas, Universidade Federal de Minas GeraisBelo Horizonte, Brazil; ^3^Laboratório de Microbiologia, Programa de Pós-graduação em Biologia Parasitária, Universidade CEUMASão Luís, Brazil; ^4^Departamento de Microbiologia, Imunologia e Parasitologia, Instituto de Ciências Biológicas e Naturais, Universidade Federal do Triângulo MineiroUberaba, Brazil

**Keywords:** *Lactobacillus rhamnosus*, *in silico* prediction, bacteriocin, BAGEL, genomic and physicochemical characterization

## Abstract

A bacteriocinogenic *Lactobacillus rhamnosus* L156.4 strain isolated from the feces of NIH mice was identified by 16S rRNA gene sequencing and MALDI-TOF mass spectrometry. The entire genome was sequenced using Illumina, annotated in the PGAAP, and RAST servers, and deposited. Conserved genes associated with bacteriocin synthesis were predicted using BAGEL3, leading to the identification of an open reading frame (ORF) that shows homology with the *L. rhamnosus* GG (ATCC 53103) prebacteriocin gene. The encoded protein contains a conserved protein motif associated a structural gene of the Enterocin A superfamily. We found ORFs related to the prebacteriocin, immunity protein, ABC transporter proteins, and regulatory genes with 100% identity to those of *L. rhamnosus* HN001. In this study, we provide evidence of a putative bacteriocin produced by *L. rhamnosus* L156.4 that was further confirmed by *in vitro* assays. The antibacterial activity of the substances produced by this strain was evaluated using the deferred agar-spot and spot-on-the lawn assays, and a wide antimicrobial activity spectrum against human and foodborne pathogens was observed. The physicochemical characterization of the putative bacteriocin indicated that it was sensitive to proteolytic enzymes, heat stable and maintained its antibacterial activity in a pH ranging from 3 to 9. The activity against *Lactobacillus fermentum*, which was used as an indicator strain, was detected during bacterial logarithmic growth phase, and a positive correlation was confirmed between bacterial growth and production of the putative bacteriocin. After a partial purification from cell-free supernatant by salt precipitation, the putative bacteriocin migrated as a diffuse band of approximately 1.0–3.0 kDa by SDS-PAGE. Additional studies are being conducted to explore its use in the food industry for controlling bacterial growth and for probiotic applications.

## Introduction

*Lactobacillus rhamnosus* is a facultative heterofermentative lactic acid bacterium (LAB) that is closely related to *Lactobacillus casei* and *Lactobacillus* zeae and encompasses a genetically diverse group of strains with a high frequency of discriminative core genome polymorphisms and a remarkable accessory genome, or variome distribution (Ceapa et al., 2015). This species has strain-specific genetic and metabolic characteristics that explain its occurrence in a variety of ecological niches (Douillard et al., 2013; Ceapa et al., 2015). Bacteriocinogenic *L. rhamnosus* strains have been isolated from human feces (Gorbach, 1996; Gill et al., 2000; Cukrowska et al., 2009; Dimitrijević et al., 2009; Aguilar-Uscanga et al., 2013), vaginal microbiota (Li et al., 2005), fermented beverages (Todorov and Dicks, 2005), grape peels (Sarika et al., 2010), milk samples (Srinivasan et al., 2013), and cheese (Jeong and Moon, 2015). This species is generally recognized as safe (GRAS) and has been widely explored as a probiotic in animal production (Weese and Anderson, 2002) human health (Gill et al., 2000; Cukrowska et al., 2009; Douillard et al., 2013; Szajewska and Kołodziej, 2015) and as a biopreservative in food systems (Cotter et al., 2005; Douillard et al., 2013).

Bacteriocins are a heterogeneous group of ribosomally synthesized peptides or proteins that have a narrow or broad antibacterial spectrum of activities against the same species or species that are phylogenetically related to the bacteriocin producer (Klaenhammer, 1993). Bacteriocin-producing strains are immune to their own bacteriocins due to the production of an immunity protein. The currently accepted system for classifying bacteriocins is based on whether they are post-translationally modified (class I) or are unmodified/minimally modified (class II) (Cotter et al., 2005, 2013).

Although the production of bacteriocins by LAB has been widely explored, few studies have been conducted using *L. rhamnosus* strains, which should be further explored due to their technological potential in human and veterinary medicines, and for food quality and safety. Since the purification of these molecules requires laborious procedures, the utilization of classical methods for new bacteriocins is cumbersome. Currently, genome mining approaches that explore both DNA and peptide databases enable prospection studies of bacteriocins *in silico* (van Heel et al., 2013). The present study reports the *in silico* prediction of bacteriocin genes in *L. rhamnosus* L156.4. Additionally, we performed *in vitro* assays to determine the antibacterial spectrum of a putative bacteriocin and made a partial physicochemical characterization.

## Materials and methods

### Isolation and characterization of the strain L156.4

Strain L156.4 was isolated from the feces of NIH mice (Taconic, Germantown, USA) and was initially identified as *L. rhamnosus* by Gram staining, catalase test, and carbohydrates fermentation pattern as determined by the API50CHL kit (bioMérieux, Marcy l'Etoile, France). The strain L156.4 was stored at −80°C in Man Rogosa Sharpe broth (MRS, Difco Laboratories Inc., Detroit, MI, USA) supplemented with 15% glycerol. Prior to the experiments, *L. rhamnosus* L156.4 was propagated twice in MRS broth for 18 h at 37°C in an anaerobic chamber (Forma Scientific Company, Marietta, OH, USA) containing an atmosphere of N_2_ (85%), H_2_ (10%), and CO_2_ (5%).

### Identification of strain L156.4 by MALDI-TOF mass spectrometry

The identification of the new strain L156.4 was performed using Matrix-Assisted Laser Desorption Ionization-Time of Flight (MALDI-TOF) Mass Spectrometry. The strain was cultured overnight on MRS agar at 37°C in anaerobic conditions. For the analysis, individual samples colonies were scraped up using a sterile plastic loop and then applied as a thin film onto a 24-spot steel plate (Bruker Daltonics, Bremen, Germany). After being air-dried, the sample was co-crystallized with 1 μl of a saturated solution of α-cyano-4-hydroxycinnamic acid matrix (HCCA; Bruker Daltonics, Bremen, Germany) in 50% acetonitrile/2.5% trifluoroacetic acid (Sigma-Aldrich, St. Louis, MO, USA). Mass spectra were acquired in reflector-positive mode on a MicroFlex LT system tabletop instrument (Bruker Daltonics) using the manufacturer's default settings. Captured spectra were analyzed using the MALDI Biotyper automation control and Bruker Biotyper 2.0 software (Bruker Daltonics, Bremen, Germany). The identification criteria used in our analysis were as follows: a score ≥2.000 indicated a species level identification, a score of 1.700 to 1.999 indicated identification at the genus level, and a score <1.700 was interpreted as not identified. *Escherichia coli* ATCC 8739 was used as a positive control.

### Next generation sequencing of genomic DNA and data analysis

The genomic DNA of *L. rhamnosus* L156.4 was extracted using a Gentra Puregene Cell kit (Qiagen, Hilden, Germany) and then was sequenced with the Illumina MiSeq Reagent kit V2 500 (http://www.illumina.com/products/miseq_reagent_kit_v2.html), using a paired end 250 prepared with the Nextera DNA Library Preparation Kit (http://www.illumina.com/products/nextera_dna_library_prep_kit.html) according to the manufacturer's recommendations. The *de novo* genome assembly was performed using the A5 pipeline (Tritt et al., 2012).

In order to infer the phylogenetic relationships of strain L156.4, the 16S rRNA gene was predicted using the software RNAmmer (Lagesen et al., 2007). The resulting 16S rDNA sequence was then searched for on NCBI using BLASTn against the 16S ribosomal RNA sequences database and the best BLAST hits were retrieved in addition to the 16S sequences from various *Lactobacillus* spp. The 16S rDNA sequence from *Lactococcus lactis* NCDO 2118 was used to root the tree (Oliveira et al., 2014). The software Muscle (Edgar, 2004) was used to generate a multiple sequence alignment and the output file was added on Splits Tree (Huson and Bryant, 2006) to create a phylogenetic tree using the Neighbor-Joining method (Saitou and Nei, 1987).

### Genome annotation, deposition, and *in silico* bacteriocin prediction

We annotated the *L. rhamnosus* L156.4 draft genome with the NCBI Prokaryotic Genome Automatic Annotation Pipeline (PGAAP) (Angiuoli et al., 2008) and Rapid Annotation Subsystem using Technology (RAST) (Aziz et al., 2008).

We used BAGEL3 (BActeriocin GEnome mining tooL), a bacteriocin search software, to predict genes related to bacteriocin synthesis, such as prebacteriocins, immunity proteins, ABC transporters, and regulation genes. The input file was the genome sequence of *L. rhamnosus* L156.4 in.fna format (van Heel et al., 2013). Afterwards, the predicted bacteriocin was submitted to a BLASTp search against the Uniprot database (http://www.uniprot.org/). In addition, conserved genes associated with bacteriocin synthesis were retrieved using the Rapid Annotation Subsystem using Technology (RAST) server (Aziz et al., 2008). The region identified in BAGEL3 and the proteins related to the predicted bacteriocin were detected and manually curated in Artemis to confirm their prediction (Rutherford et al., 2000). A search for the Pediocin-box sequence was conducted using the PFAM database. The motif YGNGVXC was used in the alignment of the predicted bacteriocin with the sequence of class IIa peptides registered on PFAM (Punta et al., 2012).

Furthermore, we made two comparisons of whole genomes using Mauve and the Artemis Comparison Tool (ACT) software in order to distinguish *L. rhamnosus* L.156.4 from *L. rhamnosus* GG (ATCC 53103) (Darling et al., 2010; Carver et al., 2005).

### Determination of the antagonistic activity

*L. rhamnosus* L156.4 was screened for its antagonistic activity using the deferred agar-spot assay and the spot-on-the lawn assay (Tagg et al., 1976). For the deferred agar-spot assay, a 5 μl sample of an 18 h MRS broth culture was spotted onto the surface of MRS agar and was incubated for 24 h at 37°C under anaerobic conditions. The cells were killed by exposure to chloroform for 30 min, and the residual chloroform was allowed to evaporate. Then, an MRS agar plate was overlaid with 3.5 ml of soft agar (0.75%) of Brain Heart Infusion (BHI) or MRS previously inoculated with indicator strains at a final concentration of 10^6^ CFU/ml (**Table 2**). Plates were then incubated for 24 h at 37°C under aerobic or anaerobic conditions according to the requirement of the indicator bacteria. The antagonistic activity was evidenced by the presence of a growth inhibition zone around the spot.

For the spot-on-the lawn assay, a total volume of 100 ml of an 18 h culture *L. rhamnosus* L156.4 in MRS broth was centrifuged at 7,500 *g* (4°C) for 15 min and the supernatant was sterilized by filtration through a 0.22-μm pore size PVDF filter (Millipore Corp., Bedford, MA, USA). An aliquot of this cell-free supernatant (CFS) was neutralized with 1 M NaOH and used as follows: a volume of 3.5 ml of MRS soft agar (0.75%) was inoculated with the strains mentioned in **Table 2** at a final concentration of 10^6^ CFU/ml. This mixture was overlaid onto MRS agar and 10 μl of the CSF was spotted directly onto this lawn. Sterile BHI or MRS media were used as a negative control. The inhibition zone was evaluated after incubation at 37°C, for 24 h in an anaerobic chamber or in aerobic conditions.

### Effect of temperature, pH, H_2_O_2_, and proteolytic enzymes on the CFS antagonistic activity

The antagonistic activity of the CFS obtained in the previous step was assessed after exposure to different pH-values, high temperatures, or in the presence of catalase and proteolytic enzymes. Aliquots of 5 ml of CFS had pH-values adjusted in a range from 3 to 9 using either sterile 1 M HCl or 1 M NaOH. Fresh MRS broth adjusted to the same pH-values was used as a control. To investigate the temperature effect on the antagonistic activity of the bacteriocin, CFS aliquots were exposed at 60, 80, and 100°C for 30 min, or at 121°C for 15 min. The samples were then allowed to cool to room temperature before being tested. The sensitivity of the antagonistic substance to enzymatic degradation by catalase and proteolytic enzymes was evaluated using catalase (E.C.1.11.1.6) at pH 7.0 (50 mM potassium phosphate buffer), trypsin (E.C.3.4.21.4, type II), α-chymotrypsin (E.C.3.4.21.1, type II), and proteinase K (E.C. 3.4.21.64) at pH 7.5 (100 mM Tris-HCl buffer), and using pepsin (E.C.3.4.23.1) at pH 3.0 (50 mM glycine buffer added at 20 mM CaCl_2_) (all enzymes were from Sigma Chemical Co., St Louis, MO, USA). Aliquots of the CFS at different pH-values were incubated (1:1 v/v) with enzyme solutions (1 mg/ml) and their respective controls at 37°C for 2 h.

After the previously mentioned treatments, the remaining antibacterial activity of the CFS was determined by spot-on-the lawn assay using the sensitive strain *Lactobacillus fermentum* ATCC 9338 at a final concentration of 10^6^ CFU/ml. This mixture was overlaid onto MRS agar, then 10 μl of each treated CFS or the respective controls were spotted directly onto the lawns. The presence of an inhibition zone was evaluated after incubation at 37°C for 24 h in an anaerobic chamber.

### Evaluation of antibacterial activity during *L. rhamnosus* l156.4 growth

*L. rhamnosus* L156.4 was used to inoculate 700 ml of 1% LAPT*g* (v/v) (Raibaud et al., 1963) and was incubated at 37°C under anaerobic conditions. Samples were removed at different time intervals for determinations of pH (model B474, Micronal, S.A., Brazil), antibacterial activity, and optical density (OD) at 600 nm, using a spectrophotometer (Biosystems Ltda, PR, Brazil). The bacterial growth was also evaluated by cell counting (CFU/ml) from aliquots of 10-fold serial dilutions in sterilized phosphate-buffered saline (PBS, pH 7.5) plated on LAPTg agar and incubated in an anaerobic chamber at 37°C for 24 h. The antibacterial activity was quantified by spotting aliquots (10 μl) of serial 2-fold dilutions of centrifuged and filtered culture medium in ultrapure water on a lawn of *L. fermentum* ATCC 9338. Arbitrary units (AU) of antagonistic activity were defined as the reciprocal of the highest serial dilution that displayed an inhibition zone and was expressed per milliliters of culture media (Tagg et al., 1976). This assay was performed in duplicate. Pearson's correlation coefficient was used to investigate the correlations between growth and the putative bacteriocin production. Values of *p* < 0.05 were considered statistically significant.

### Partial purification of the putative bacteriocin by precipitation

*L. rhamnosus* L156.4 was cultivated in LAPTg broth (100 ml) for 18 h in an anaerobic chamber. The CFS, obtained as described in section Effect of Temperature, pH, H_2_O_2_ and proteolytic enzymes on the CFS antagonistic, activity was precipitated in an ice bath with ammonium sulfate to 40% saturation, and then centrifuged (12,500 *g*, 30 min, 4°C). The pellet was resuspended in 5 ml of ammonium acetate buffer (50 mM, pH 5.0), and desalted against ultrapure Milli-Q water using a 1 kDa cut-off dialysis membrane (Spectrum Inc., CA, USA). The desalted CFS (DCFS) and the same volume of CFS were freeze-dried. Then, the powder was dissolved in 50 μl of ultra-pure water, and the inhibitory activity of this fraction was determined by a spot-on-the lawn assay using *L. fermentum* ATCC 9338 as the indicator strain (Tagg et al., 1976).

### Direct detection of the putative bacteriocin on gels

In order to estimate the molecular mass of the bacteriocin, we estimated the position of the inhibitory zone of CFS and DCFS in the gel. Aliquots of CFS and DCFS were subjected to Tricine-sodium dodecyl sulfate-polyacrylamide gel electrophoresis (Tricine-SDS-PAGE) as described by Schägger and Von Jagow (1987) using a 16.5% gel. After electrophoresis at 60 mA for 3 h, the gel was cut into two vertical sections. Half of the gel was stained with Coomassie Brilliant blue R250 (Bio-Rad, Hercules, CA, USA) and the other half was fixed for 2 h in a 20% 2-propanol/10% acetic acid solution, and then was extensively washed with regularly replaced sterile water for 6 periods of 30 min. To detect the bacteriostatic region, the gel was overlaid with LAPTg soft agar (0.75%), seeded with *L. fermentum* ATCC 9338 as the indicator strain. After an overnight incubation at 37°C, the gel was examined for the presence of inhibition zones. The molecular mass of the bacteriocin was estimated by a relative mobility method, comparing the migration pattern of the bacteriocin to a mixture of protein markers (ultra-low molecular weight marker M3546, Sigma-130 Aldrich, St. Louis, MO, USA; Bhunia et al., 1987).

## Results

### Microbial identification and phylogenetic tree

**Figure 1:**
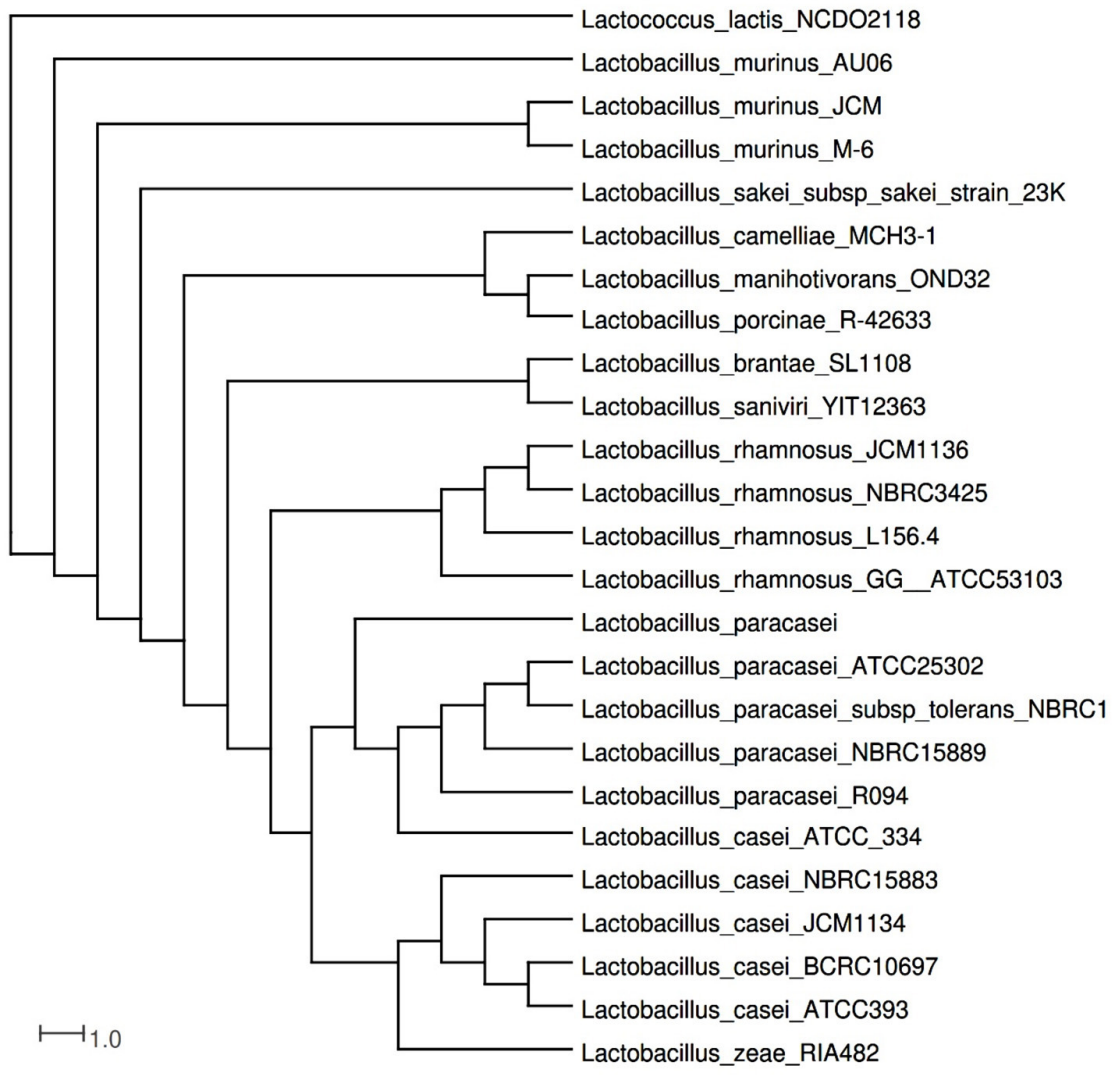
**Phylogenetic tree of *L. rhamnosus* LL156.4 obtained by a Neighbor-Joining alignment of 1,567 nucleotide positions in 16S rDNA and compared to 234 *Lactobacillus* spp. sequences**. *L. rhamnosus* strain LL156.4 was positioned among *L. rhamnosus* NBRC3425 and *L. rhamnosus* JCM1136.

The identity of *L. rhamnosus* L156.4 was determined by both MALDI-TOF MS analyses and DNA sequencing. By comparing the 16S rDNA sequences (accession number KX644947) with other *L. rhamnosus* strains deposited in GenBank, the identification of the strain was confirmed as *L. rhamnosus*, with an identity threshold >98%. Phylogenetic inferences confirmed the identification of the L156.4 strain as *L*. *rhamnosus*, which was most closely related to the *L. rhamnosus* JCM1136 and NBRC3425 strains (Figure 1).

### Genomic characterization and prediction of the putative bacteriocin

The Whole Genome Shotgun project was deposited at DDBJ/ENA/GenBank (https://www.ncbi.nlm.nih.gov/genbank/) under the accession MTJY00000000. The version described in this paper is version MTJY01000000. We predicted the presence of an ORF (AOI_1; orf010: locus tag BWR10_11520) using BAGEL3 and PGAAP. This region presented 100% identity with the prebacteriocin of *L. rhamnosus* strain GG (ATCC 53103) sequence WP_005686837.1 with an *E*-value of 3.9e-05 and 113 amino acids (Table 1). The scanning of this sequence for conserved motifs, as described at http://pfam.sanger.ac.uk, indicated the presence of a putative conserved domain belonging to the Enterocin A superfamily (pfam08951). The Enterocin A domain predicted in orf010 is incomplete, and it corresponds to amino acids 12–66 of the full sequence. A pediocin-like YGNGVXC motif, characteristic of class IIa bacteriocins, was not found in the genome of *L. rhamnosus* 156.4.

**Table 1:** ***In silico* prediction of the functions of proteins associated with bacteriocin synthesis from *Lactobacillus rhamnosus* L156.4**.

**Locus tag**	**Predicted protein function (PGAAP)**	**Length (aa)**	**Protein deposit number**	**Protein accession number (BLASTp)/homology screening**
BWR10_11430	Bacteriocin ABC transporter permease	459	KY355786	WP_031547267.1/*L. rhamnosus* HN001
BWR10_11435	Bacteriocin cleavage/export ABC transporter	730	KY355785	WP_005686870.1/*L. rhamnosus* HN001
BWR10_11440	ATP-binding protein	431	KY355784	WP_005686867.1/*L. rhamnosus* HN001
BWR10_11445	DNA-binding response regulator	258	KY355783	WP_005686865.1/*L. rhamnosus* HN001
BWR10_11450	Hypothetical protein	69		
BWR10_11455	Hypothetical protein	60	ND	
BWR10_11460	Hypothetical protein	66		
BWR10_11465	Hypothetical protein	81	KY355782	
BWR10_11470	Bacteriocin immunity protein	99	KY355781	WP_031546828.1/*L. rhamnosus* HN001
BWR10_11475	Bacteriocin leader domain-containing protein	52		
BWR10_11480	Hypothetical protein	75		
BWR10_11485	Bacteriocin leader domain-containing protein	61	ND	
BWR10_11490	Hypothetical protein	61		
BWR10_11495	Hypothetical protein	61		
BWR10_11500	CAAX protease family protein	268	KY355779	WP_005686845.1/*L. rhamnosus* HN001
BWR10_11505	Rrf2 family transcriptional regulator	146	KY355778	WP_005686843.1/*L. rhamnosus* HN001
BWR10_11510	MFS transporter	475	KY355777	WP_005686841.1/*L. rhamnosus* HN001
BWR10_11515	Hypothetical protein	110	KY355776	
BWR10_11520	Bacteriocin immunity protein^*^	113	KY355775	WP_005686837.1/*L. rhamnosus* GG
BWR10_11525	Aldo/keto reductase	317	KY355774	
BWR10_11530	Alpha-galactosidase	81	ND	
BWR10_11535	Alpha-galactosidase	57		
BWR10_11540	Oxidoreductase	244	KY355772	
BWR10_11545	NADH-dependent flavin oxidoreductase	381	KY355771	
BWR10_11550	Hypothetical protein	105	KY355770	
BWR10_11555	Class II fumarate hydratase	459	KY355769	

*ND: sequences were not deposited*.

* *Detected in RAST, PGAAP and BAGEL3*.

**Figure 2:**
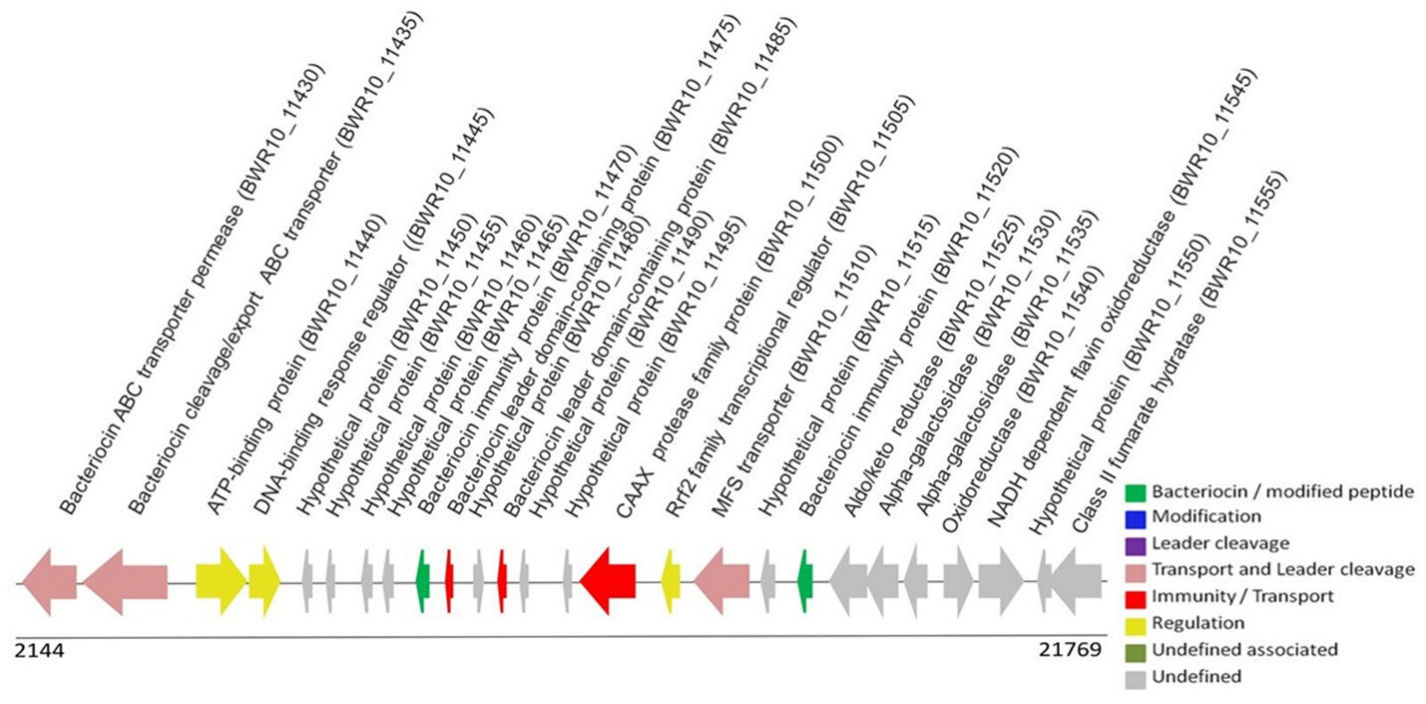
**BAGEL3 graphical output for putative prebacteriocin (orf010 in green—locus tag: BWR10_11520)**. The manual annotation of the predicted locus tag in PGAAP required for bacteriocin synthesis is identified according to the legend.

Furthermore, genes that encode components required for bacteriocin synthesis, regulation and hypothetical proteins were detected in the genome of *L. rhamnosus* L156.4 and are shown in Figure 2. All predicted locus tags were manually annotated in order to check and confirm the predicted information. The accession numbers of the coding sequences are shown at Table 1.

Considering the similarities of the bacteriocins of *L. rhamnosus* L156.4 and *L. rhamnosus* GG (ATCC 53103), we conducted a comparative analysis of the whole genomes of these strains. The results showed that the related region (cluster) of the predicted bacteriocin of *L. rhamnosus* L156.4 is not identical to the one identified in *L. rhamnosus* GG (ATCC 53103). Using both approaches (Mauve and ACT), it is possible to see some differences in the genomes regarding regions of deletions and insertions (Supplementary Figures S1, S2).

### Spectrum of antibacterial activity of antagonistic substances

The deferred agar-spot assay showed the inhibitory activity of antimicrobial substances produced by *L. rhamnosus* L156.4 against enteropathogenic *E. coli, Bacillus cereus, Staphylococcus aureus, Listeria monocytogenes* and other gram-positive and gram-negative bacteria, but not against *Lactobacillus acidophilus, Lactobacillus brevis* and *Lactobacillus rhamnosus*. Additionally, the spot-on-the-lawn assay was performed using the neutralized CFS, and no inhibition zone was observed after this treatment for all gram-negative indicator strains, suggesting the inhibitory effect was due in part to the action of organic acids (Table 2).

**Table 2:** **Antibacterial activity spectrum of *Lactobacillus rhamnosus* LL156.4 in the deferred agar-spot and spot-on-the lawn assays**.

**Indicator strain**	**Source**	**Deferred agar-spot assay^f^**	**Spot-on-the lawn assay^g^**
*Escherichia coli*	ATCC^a^ 5723	+^d^	−
Enteropathogenic *E. coli* (EPEC)	CDC^b^ O111ab	+	−
Enterohemorrhagic *E. coli* (EHEC) Enteroinvasive *E. coli* (EIEC)	ATCC 43895 ATCC 43893	+ +	−−
Enterotoxigenic *E. coli* (ETEC)	H10407	+	−
Enteroaggregative *E. coli* (EAEC)	042	+	−
*Klebsiella pneumoniae*	ATCC 13822	+	−
*Salmonella enterica* serovar Typhimurium	ATCC 13311	+	−
*Shigella sonnei*	ATCC 11060	+	−
*Listeria monocytogenes*	ATCC 15313	+	+
*Listeria monocytogenes*	ATCC 19115	+	+
*Listeria monocytogenes*	ATCC 6477	+	+
*Listeria monocytogenes*	Scott A	+	+
*Corynebacterium fimi*	NCTC^c^7547	+	+
*Micrococcus luteus*	ATCC 49732	+	+
*Staphylococcus aureus*	ATCC 29213	+	+
*Bacillus cereus*	ATCC 11778	+	+
*Enterococcus faecalis*	ATCC 19433	+	+
*Lactobacillus acidophilus*	ATCC 4356	−^e^	−
*Lactobacillus brevis*	ATCC 367	−	−
*Lactobacillus rhamnosus*	ATCC 7469	−	−
*Lactobacillus delbrueckii* subsp. *lactis*	ATCC 7830	+	+
*Lactobacillus fermentum*	ATCC 9338	+	+
*Lactobacillus plantarum*	ATCC 8014	+	+

a *ATCC, American Type Culture Collection, Rockville, MD, USA*.

b *CDC, Center for Diseases Control, Atlanta, GA, USA*.

c NCTC, National Collection of Type Cultures, Central Public Health Laboratory, London, UK

d Presence of inhibition zone (+)

e Absence of inhibition zone (−)

g *Assay conducted with cells of L156.4*.

h *Assay conducted with the cell-free supernatant (CFS) at pH 7*.

### Physicochemical characterization of antagonistic substances

The effect of temperature, pH, H_2_O_2_ and proteolytic enzymes on the antibacterial activity of CSF was evaluated. The inhibitory activity of CSF against *L. fermentum* ATCC 9338 was maintained at pH-values ranging from 3 to 9 and was not altered by heat treatment after 30 min at 60, 80, 100, or 121°C. Moreover, the inhibitory activity was observed after catalase treatment, but not after being treated with proteolytic enzymes (Table 3).

**Table 3:** **Effect of temperature, pH, H_2_O_2_ and proteolytic enzymes on the antagonistic activity against *L. fermentum* ATCC 9338**.

**Treatments**	**Inhibitory activity of CFS^a^**
**pH**	
3.0	+^a^
4.0	++^b^
5.0	++
6.0	++
7.0	++
8.0	+
9.0	+
**ENZYMES**	
Catalase	++
α-Chymotrypsin	−^c^
Proteinase K	−
Trypsin	−
Pepsin	−
**CONTROLS**	
MRS broth	−
CFS pH 3.0	+
CFS pH 4.2	++
CFS pH 7.5	++
Potassium phosphate 50 mM; pH 7.0	−
Tris-HCl 100 mM; pH 7.5	−
Glycine added at 20 mM CaCl_2_, (50 mM; pH 3.0)	−
**TEMPERATURE**	
Control (25°C)	++
60°C, 30 min	++
80°C, 30 min	++
100°C, 30 min	++
121°C, 15 min	++

a *Presence of inhibition zone with growth of sparse colonies (+)*.

b *Presence of clear inhibition zone (++)*.

c *Absence of inhibition zone (−)*.

### Production of putative bacteriocin in LAPTg medium

**Figure 3:**
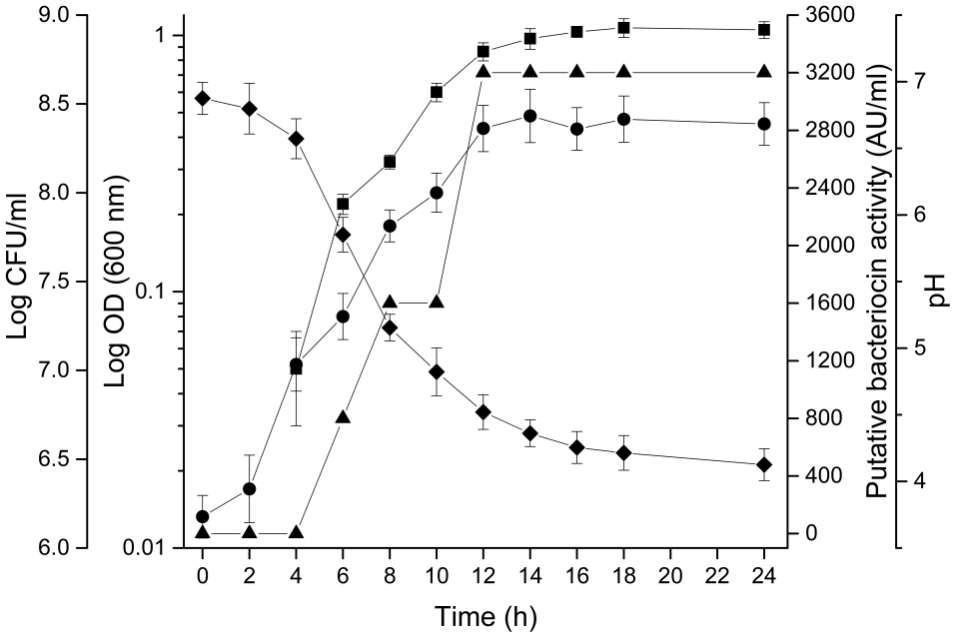
**Evaluation of production of putative bacteriocin in culture medium**. Growth curve of L. *rhamnosus* LL156.4 in LAPT*g* broth at 37°C in anaerobic conditions. Symbols: optical density (OD) 600 nm (•), Log CFU/ml (■), pH (♦), putative bacteriocin activity (AU/ml) (▴).

Figure 3 shows the growth curve of *L. rhamnosus* L156.4 in LAPTg broth. The increase in bacterial counting, as determined by CFU counting and optical density, was accompanied by pH decrease from 6.8 to 4.0 after 24 h. The production of the antibacterial substances started after 4 h of incubation and occurred during logarithmic growth phase, reaching a maximum value of 3,200 AU/ml after 12 h and was constant for up to 24 h. In addition, a positive correlation was observed between the variables, indicating that the production of substances is associated with bacterial growth (*r*^2^ = 0.94, *p* < 0.05 for log CFU/ml and *r*^2^ = 0.98, *p* < 0.05 for OD at 600 nm).

### Partial purification of the putative bacteriocin by salt precipitation

The putative bacteriocin in the CFS was partially purified by a 40% salt saturation precipitation, and its antibacterial activity against *L. fermentum* remained stable after desalting with a 1 kDa cut-off membrane. The supernatant showed no antibacterial activity after removing precipitated proteins.

### SDS-PAGE analysis and direct detection of the putative bacteriocin on gels

**Figure 4:**
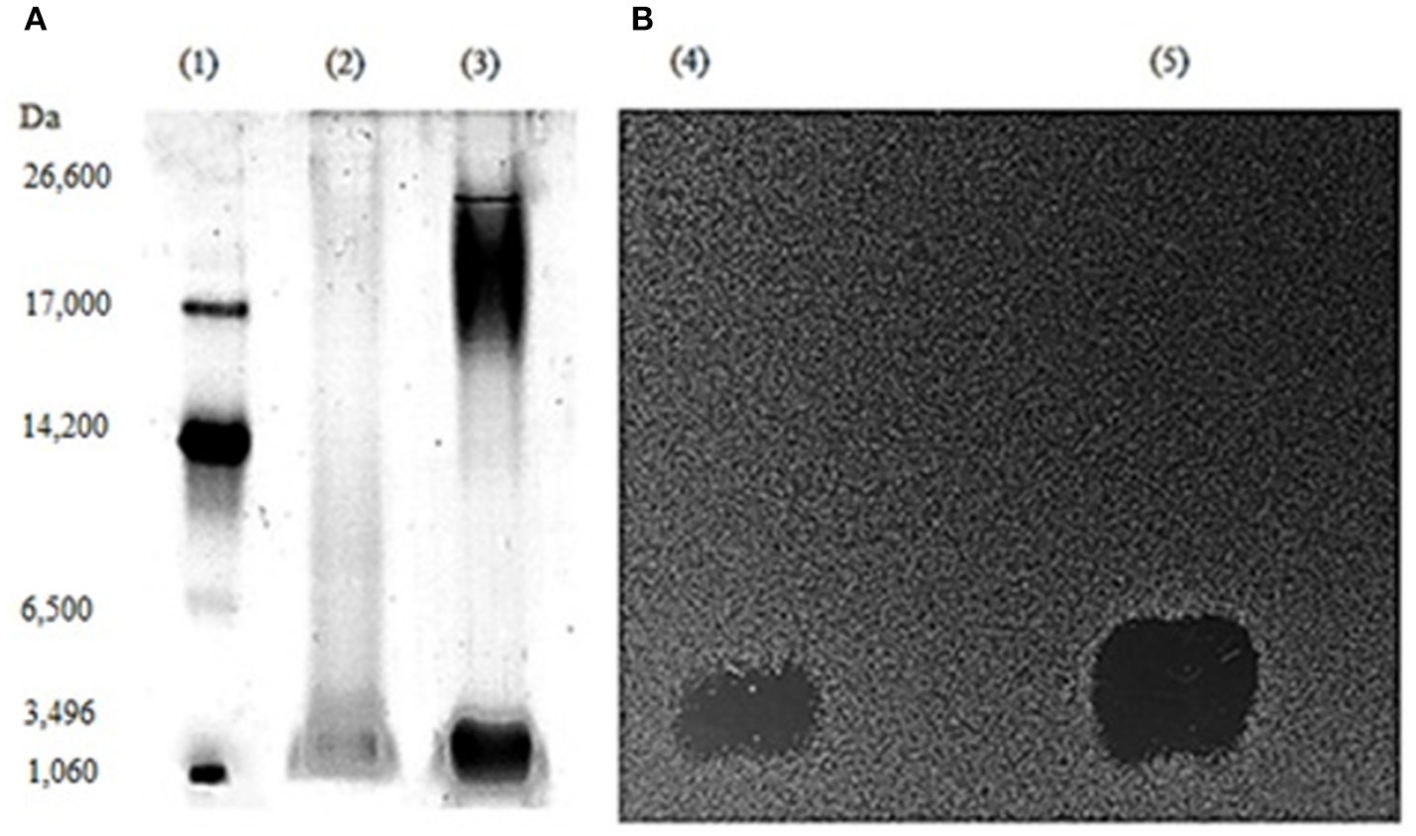
**Molecular mass evaluation by simultaneous visual detection of bacteriocin activity by Tricine SDS-PAGE. (A)** Gel stained with Comassie Brilliant Blue R250: lanes 1, 2, and 3 represent molecular weight standards, cell free supernatant (CFS), and desalted ammonium sulfate precipitated supernatant (DCFS), respectively. **(B)** Gel overlaid with MRS soft agar inoculated with *L. fermentum* ATCC 9338: lanes 4 and 5 represent CFS and DCFS, respectively.

The examination of the protein profile by Tricine SDS-PAGE stained with Coomassie Blue (Figure 4A, lanes 2, and 3), revealed a diffuse band of approximately 1.0–3.0 kDa for both CFS and DCFS (Figure 4A, lanes 2, and 3), which coincided with a single zone of bacterial inhibition for both CFS and DCFS (Figure 4B, lanes 4, and 5). The results also showed an increase of the band and inhibition zone size corresponding to the active compound in the DCFS when compared to the CFS, for both Coomassie Blue staining and inhibitory activity.

## Discussion

Data mining of genomic and metagenomic sequences has been an important strategy for the identification of bacteriocin producers. This is a promising approach since many features of bacteriocin gene clusters, and especially bacteriocin modification genes, are highly conserved. The synthesis of class II bacteriocins is dependent on the expression of at least four genes that are organized in one or two operons, with relevant genes including: the prebacteriocin structural gene, an immunity protein-associated gene, a gene encoding an ABC-transporter that exports the bacteriocin simultaneous with the processing of the leader sequence, and a gene encoding an accessory protein whose function remains unknown (Drider et al., 2006).

We annotated the whole genome using PGAAP, which uses a combination of gene prediction methods through a Hidden Markov Model (HMM) with an approach based on sequence similarity (Angiuoli et al., 2008). Moreover, we used the web-based software BAGEL3 for *in silico* prospection of class I, II and III bacteriocins through a knowledge-based bacteriocin database and motif databases (van Heel et al., 2013). Using this tool, we provided evidence of a putative bacteriocin produced by *L. rhamnosus* L156.4 isolated from the feces of NIH mice, which was further confirmed by *in vitro* assays. Reports on bacteriocins produced by *L. rhamnosus* remain scarce. Strains of this species have been isolated from the intestinal tract of humans and animals (Heilig et al., 2002) and to the best of our knowledge, bacteriocinogenic *L. rhamnosus* fecal strains have been detected only in human feces (Gorbach, 1996; Gill et al., 2000; Cukrowska et al., 2009; Dimitrijević et al., 2009; Aguilar-Uscanga et al., 2013). Thus, this is the first report of a putative bacteriocin produced by a *L. rhamnosus* strain (L156.4) that was obtained from the feces of an NIH mouse.

Using BAGEL3, we found that the ORF of the prebacteriocin in *L. rhamnosus* L156.4 showed 100% identity with that of *L. rhamnosus* GG (ATCC 53103), a probiotic strain (Szajewska and Kołodziej, 2015) commercialized by Chr. Hansen (Hørsholm, Denmark). Previous investigations with *L. rhamnosus* GG described an 8.7-Kb putative type IIb bacteriocin operon, which includes an exporter protein, an ABC/C39-type peptidase, a two-component signal transduction system, an immunity protein and the bacteriocin gene (Kankainen et al., 2009). PGAP, RAST, and BLASTp analyses allowed the detection of other ORFs that encoded proteins such as prebacteriocin (98 amino acids), an immunity protein, ABC transporter proteins, and regulatory proteins sharing 100% similarity with ORFs of *L. rhamnosus* HN001, a probiotic strain (Gill et al., 2000) commercialized by Danisco (DuPont, Las Vegas, NV, USA). Both predicted putative bacteriocins contain the Enterocin A domain (pfam08951), but the regions are different, and one of them is incomplete (orf010). Due to the similarities of our putative bacteriocin to that of *L. rhamnosus* GG (ATCC 53103), we conducted a comparative analysis of the whole genomes of these strains. In spite of the great synteny between the genes related to the putative bacteriocin, the cluster of the predicted bacteriocin in *L. rhamnosus* L156.4 is not identical to the one identified in *L. rhamnosus* GG (ATCC 53103). Moreover, the phylogenetic tree showed that *L. rhamnosus* L156.4 is more closely related to *L. rhamnosus* JCM1136 and NBRC3425 strains.

A search for the YGNGVXC motif, a characteristic of class IIa bacteriocins, was conducted in the *L. rhamnosus* L156.4 genome, but this pediocin-like sequence was not found. However, the CAAX amino terminal protease of self-immunity, which is indicative of class IIb bacteriocins, was detected (Pei and Grishin, 2001). Nevertheless, it is important to mention that our data were obtained from the analysis of a draft genome, and thus, it is possible that some information related to the bacteriocin is missing. More studies and analyses using a complete genome are being planned.

The antibacterial activity spectrum was evaluated using the deferred agar-spot and spot-on-the lawn assays (Table 2). Among gram-positive target strains, the inhibition of *Enterococcus faecalis, L. fermentum, Lactobacillus delbrueckii* subsp. *lactis, Lactobacillus plantarum*, and *L. monocytogenes* can be attributed to the putative bacteriocin, considering that the mentioned antibacterial spectrum and antilisterial activity are among the main features of class II bacteriocins produced by LAB (Klaenhammer, 1993; Drider et al., 2006). Our results present some overlapping features with other reports of antimicrobial activity of bacteriocins produced by *L. rhamnosus* strains against *E. faecalis* (Todorov and Dicks, 2005; Aguilar-Uscanga et al., 2013), *Micrococcus luteus* (Srinivasan et al., 2013), *S. aureus* (Sarika et al., 2010; Srinivasan et al., 2013; Jeong and Moon, 2015), *L. monocytogenes* (Aguilar-Uscanga et al., 2013; Srinivasan et al., 2013; Jeong and Moon, 2015) and *E. coli* (Todorov and Dicks, 2005). Class II bacteriocins kill bacteria by pore formation or by interfering with the integrity of the target cell membrane, inducing permeabilization and leakage of the intracellular content (Drider et al., 2006).

Bacteriocins can be effective against gram-negative bacteria, but this effect is limited due to the protective effect of the outer membrane (Helander et al., 1997; Cotter et al., 2005). Here, antagonism against the gram-negative enteropathogenic bacteria *E. coli, Salmonella* Typhimurium, *Shigella sonnei*, and *Klebsiella pneumoniae* can be the production of organic acids by *L. rhamnosus* L156.4, which is a facultatively heterofermentative species. The neutralization of the pH of the supernatant confirmed an effect of organic acids on pH decrease. The antimicrobial activities of the bacteriocin, organic acids, and the acidic pH are complementary and might be synergistic (Helander et al., 1997). The antimicrobial mechanism of these acids is mostly associated with their ability to cross the cytoplasmic membrane in its un-disassociated form, resulting in reduced intracellular pH and the disruption of the transmembrane proton motive force, particularly in gram-negative bacteria (Alakomi et al., 2000). Moreover, it has been demonstrated that membrane permeabilization by lactic acid can potentiate the effect of antimicrobial peptides, suggesting a synergic behavior of these compounds (Niku-Paavola et al., 1999).

Physicochemical characterization assays were performed with the CFS of *L. rhamnosus* L156.4 using *L. fermentum* ATCC 9338 as the indicator species (Table 3). The putative antimicrobial compound present in the CSF was heat-resistant and remained active at pH-values ranging from 3 to 9, and it remained stable at all tested temperatures. In addition, the CSF lost its activity after treatment with proteases, confirming its proteinaceous nature, indicating that *L. rhamnosus* L156.4 is a bacteriocin-producer strain. The possibility of the inhibitory effect observed against the indicator strain being caused by hydrogen peroxide was discarded, given that the producer strain was cultured anaerobically and that the antibacterial effect was not altered after treatment with catalase.

The physicochemical characteristics described in this study for the inhibitory product of *L. rhamnosus* L156.4 had also been observed for other bacteriocins. Rhamnosin A is a small non lanthionine-containing bacteriocin produced by *L. rhamnosus* strain 68, which also retained its biological activity after thermal treatment (95°C, 30 min) and was sensitive to the proteolytic activity of pepsin and trypsin (Dimitrijević et al., 2009). Similarly, the bacteriocin produced by *L. rhamnosus* GP1 was stable at pH-values ranging from 2.5 to 8.5, and after autoclaving at 121°C for 20 min (Sarika et al., 2010).

Previous reports have demonstrated the influence of the culture medium composition on bacterial growth and production of antimicrobial compounds. Although MRS medium is generally used for antagonism assays and physicochemical characterization, LAPTg broth was chosen for evaluation of bacteriocin production and partial purification because it contains lower amounts of potentially interfering proteins or peptides than does MRS. The same medium was used in other studies for bacteriocin purification (Ocaña et al., 1999; Tomás et al., 2002). Tomás et al. (2002) used a complete factorial design to compare the production of bacteriocin by *L. salivarius* subsp. *salivarius* CRL 1328 in LAPTg, and in an initial pH of 6.5, its maximum bacteriocin activity (1,280 AU/ml) was detected after a 6 h incubation at 37°C. In similar conditions, we observed the production of 3,200 AU/ml after a 12 h incubation at 37°C, and confirmed a positive correlation between the bacterial growth and the putative bacteriocin production. This pattern was already described for other lactic acid bacteria (Ocaña et al., 1999; Tomás et al., 2002). Nevertheless, Todorov and Dicks (2005) detected a high level of bacteriocin production (12,800 AU/ml) by *L. rhamnosus* strains ST461BZ and ST462BZ culture in MRS medium after a 15 h of incubation at 30°C.

The protein profile examined on a SDS-PAGE-Tricine gel showed a diffuse band of equal mobility pattern (1–3 kDa) in both samples CSF and DCFS samples, which presented an antagonistic activity *in situ* against the indicator strain (Figure 4). As estimated by the same method, other bacteriocins produced by *L. rhamnosus* strains showed molecular sizes, ranging from 2.8 to 8.0 kDa (Li et al., 2005; Todorov and Dicks, 2005; Aguilar-Uscanga et al., 2013; Srinivasan et al., 2013). Molecular masses of 6433.8 and 6,502 Da were obtained by mass spectrometry analyses of rhamnosin A (Dimitrijević et al., 2009) and a bacteriocin described by Yue et al. (2013), respectively.

## Conclusions

A putative bacteriocin produced by *L. rhamnosus* L156.4 was predicted *in silico* and inhibited the growth of several bacteria *in vitro*, including gram-positive human and foodborne bacterial pathogens. Its antilisterial activity supports further studies in order to explore it for food preservation and for use as a probiotic.

## Author contributions

RN and JN designed the study and drafted the manuscript. AS performed *in vitro* experiments. VA, SS, LO, and AM performed next generation sequencing and *in silico* analyses. RN, MD, AM, and Vd analyzed the results and wrote the manuscript. RN and MD reviewed the final version of the manuscript. All authors read and approved the manuscript after contributing with suggestions.

### Conflict of interest statement

The authors declare that the research was conducted in the absence of any commercial or financial relationships that could be construed as a potential conflict of interest.

## Supplementary material

Supplementary Figure S1 **Gene synteny between *L. rhamnosus* GG (ATCC 53103) and *L. rhamnosus* L156.4**. *Lactobacillus rhamnosus* GG (ATCC 53103) (top) was used as a reference for the comparison analysis. The genomes are represented according to the nucleotide conservation and synteny. Low similarity regions are represented as white regions inside the blocks, highlighted by a black (^*^). Regions of deletions are represented as blank spaces between the blocks and by the letter **(A)**, and an inversion region are represented by letter **(B)**. To perform the genome synteny analysis, we used the software Mauve, which compares the genomes by identifying and clustering homologous genes between the genomes into large collinear blocks of genes.

Supplementary Figure S2 **ACT comparison between *L. rhamnosus* GG (ATCC 53103) and *L. rhamnosus* L156.4**. On top, the complete genome of *L. rhamnosus* GG (ATCC 53103); on bottom, the contig of *L. rhamnosus* L156.4 that contains the predicted bacteriocin. Regions of similarity between the two genomes are marked in red; the bacteriocin is highlighted in yellow and is present in both genomes (green rectangle). In addition, regions of deletions and insertions are represented by blank spaces.
