# Supplementary figures and images for: In silico Prediction, in vitro Antibacterial Spectrum, and Physicochemical Properties of a Putative Bacteriocin Produced by Lactobacillus rhamnosus Strain L156.4
Source: Front Microbiol. 2017 May 19;8:876. doi: 10.3389/fmicb.2017.00876 (PMC5437165; doi:10.3389/fmicb.2017.00876)

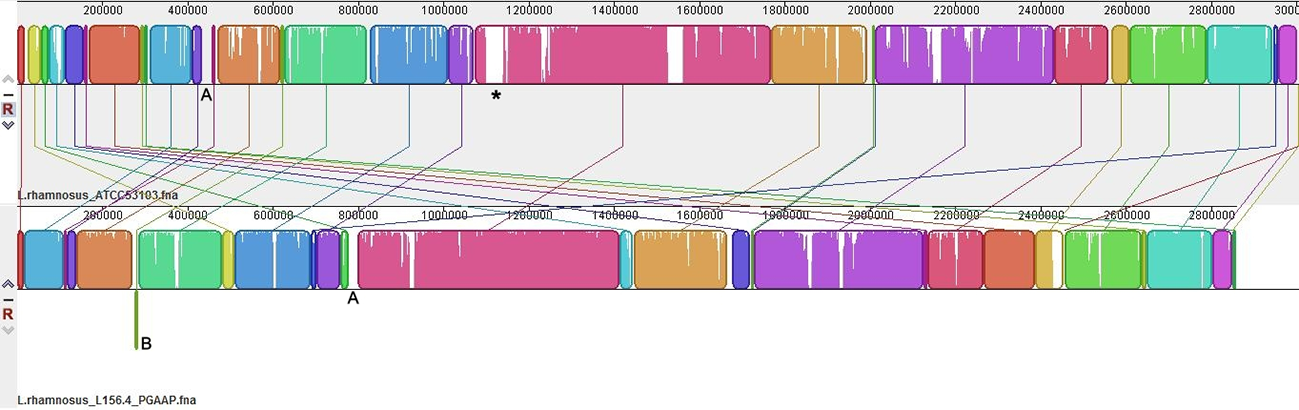

Supplement: Supplementary Figure S1 — Gene synteny between L. rhamnosus GG (ATCC 53103) and L. rhamnosus L156.4. Lactobacillus rhamnosus GG (ATCC 53103) (top) was used as a reference for the comparison analysis. The genomes are represented according to the nucleotide conservation and synteny. Low similarity regions are represented as white regions inside the blocks, highlighted by a black (*). Regions of deletions are represented as blank spaces between the blocks and by the letter (A), and an inversion region are represented by letter (B). To perform the genome synteny analysis, we used the software Mauve, which compares the genomes by identifying and clustering homologous genes between the genomes into large collinear blocks of genes. [file Image1.TIF]

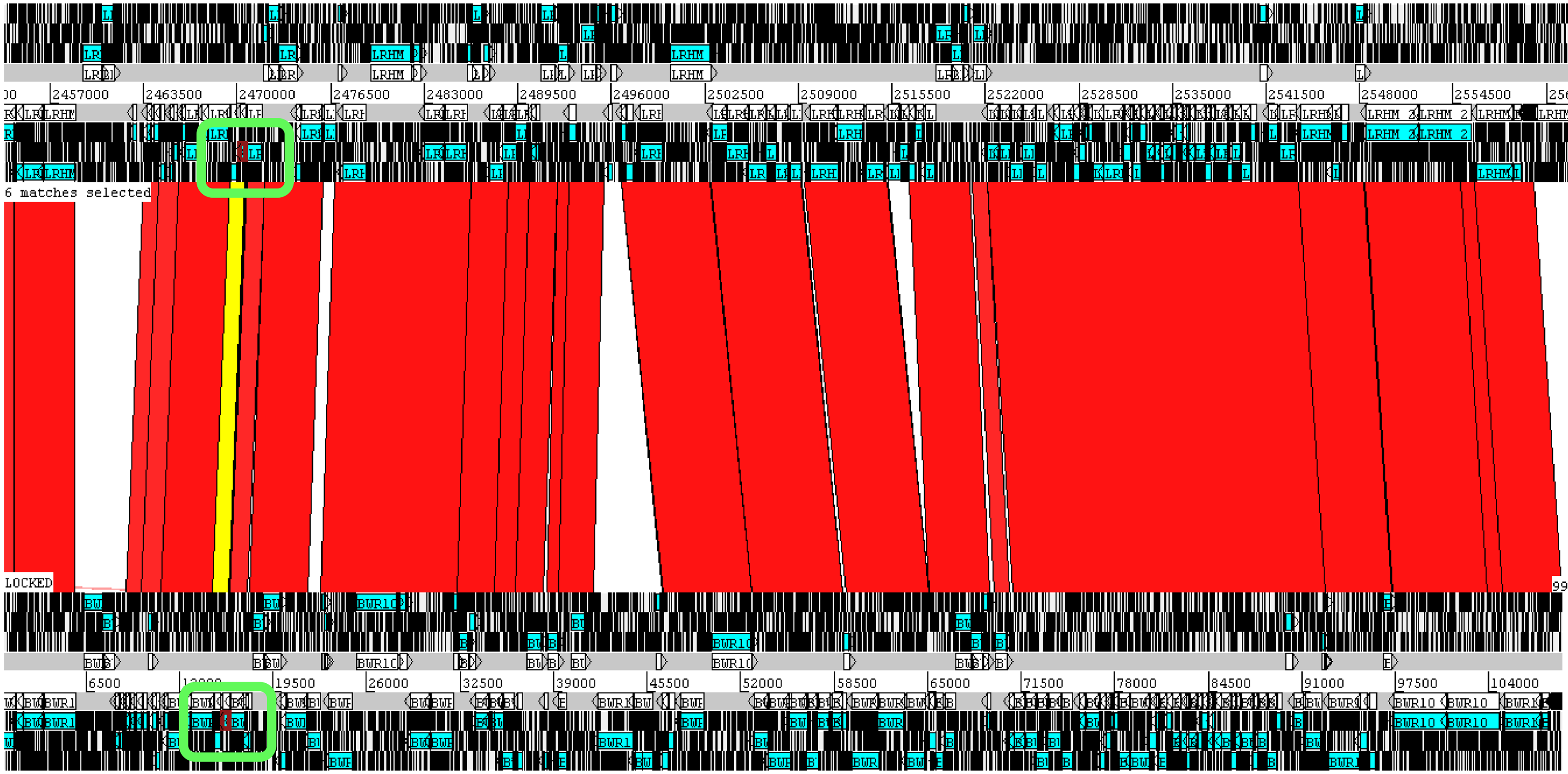

Supplement: Supplementary Figure S2 — ACT comparison between L. rhamnosus GG (ATCC 53103) and L. rhamnosus L156.4. On top, the complete genome of L. rhamnosus GG (ATCC 53103); on bottom, the contig of L. rhamnosus L156.4 that contains the predicted bacteriocin. Regions of similarity between the two genomes are marked in red; the bacteriocin is highlighted in yellow and is present in both genomes (green rectangle). In addition, regions of deletions and insertions are represented by blank spaces. [file Image2.TIF]
